# Highly active engineered-enzyme oriented monolayers: formation, characterization and sensing applications

**DOI:** 10.1186/1477-3155-9-26

**Published:** 2011-06-20

**Authors:** Abraham Ulman, Michael Ioffe, Fernando Patolsky, Elisha Haas, Dana Reuvenov

**Affiliations:** 1 Department of Chemistry, Bar-Ilan University, Ramat-Gan 52900, Israel; 2 Department of Chemistry, Tel-Aviv University, Tel Aviv 69978, Israel; 3 Faculty of Life Sciences, Bar-Ilan University, Ramat-Gan 52900, Israel; 4 Department of Chemical and Biological Sciences, Polytechnic Institute of NYU, Six Metrotech Centre, Brooklyn, NY 11201, USA

## Abstract

**Background:**

The interest in introducing ecologically-clean, and efficient enzymes into modern industry has been growing steadily. However, difficulties associated with controlling their orientation, and maintaining their selectivity and reactivity is still a significant obstacle. We have developed precise immobilization of biomolecules, while retaining their native functionality, and report a new, fast, easy, and reliable procedure of protein immobilization, with the use of *Adenylate kinase *as a model system.

**Methods:**

Self-assembled monolayers of hexane-1,6-dithiol were formed on gold surfaces. The monolayers were characterized by contact-angle measurements, Elman-reagent reaction, QCM, and XPS. A specifically designed, mutated *Adenylate kinase*, where cysteine was inserted at the 75 residue, and the cysteine at residue 77 was replaced by serine, was used for attachment to the SAM surface *via *spontaneously formed disulfide (S-S) bonds. QCM, and XPS were used for characterization of the immobilized protein layer. Curve fitting in XPS measurements used a Gaussian-Lorentzian function.

**Results and Discussion:**

Water contact angle (65-70°), as well as all characterization techniques used, confirmed the formation of self-assembled monolayer with surface SH groups. X-ray photoelectron spectroscopy showed clearly the two types of sulfur atom, one attached to the gold (triolate) and the other (SH/S-S) at the ω-position for the hexane-1,6-dithiol SAMs. The formation of a protein monolayer was confirmed using XPS, and QCM, where the QCM-determined amount of protein on the surface was in agreement with a model that considered the surface area of a single protein molecule. Enzymatic activity tests of the immobilized protein confirmed that there is no change in enzymatic functionality, and reveal activity ~100 times that expected for the same amount of protein in solution.

**Conclusions:**

To the best of our knowledge, immobilization of a protein by the method presented here, with the resulting high enzymatic activity, has never been reported. There are many potential applications for selective localization of active proteins at patterned surfaces, for example, bioMEMS (MEMS - Micro-Electro-Mechanical Systems. Due to the success of the method, presented here, it was decided to continue a research project of a biosensor by transferring it to a high aspect ratio platform - nanotubes.

## Introduction

The interest in introducing ecologically-clean, and efficient enzymes into modern industry has been growing steadily, because of their high specificity and activity [1-6]. Proteins are biological machines, and many of them preserve their stable structure under harsh conditions. In the past, we immobilized *Candida rugosa *lipase (E.C.3.1.1.3) on *γ*-Fe_2_O_3 _magnetic nanoparticles [7]. However, while we have observed constant activity over one month, the activity of the enzyme was only 1% of that in solution. We speculated that the observed low reactivity must result from the protein's conformation in the immobilized state, or possibly because reactants in solution have limited access to the active site.

The mechanistic consequences of protein adsorption on a surface can be studied at the molecular level with the use of self-assembled monolayers (SAMs) [8-11] as intermediate layers, where one has control of surface chemical functionalities and their densities. SAMs are ordered, close-packed, single layers of molecules on substrates, formed by spontaneous organization of surface-active molecules. These organic interfaces, which have properties largely controlled by the end groups of the adsorbates, provide ample opportunities for technologies that seek to exploit their adaptable character. Such surface-engineering capabilities, when combined with proteins, can result in highly-diverse systems, in which the environment of the attached protein can be designed to be close to that existing in nature.

There are many potential applications for selective localization of active proteins at specific sites of patterned surfaces, for example, bioMEMS (MEMS - Micro-Electro-Mechanical Systems). However, the study and application of proteins have been challenged by the inherent difficulties associated with controlling their orientation in the immobilized state, and maintaining the high selectivity and reactivity of immobilized proteins has remained a significant obstacle. Overcoming this obstacle, and developing precise immobilization of biomolecules in well-defined patterns, while retaining their native functionality and activity, will be an important enabling technology. Therefore, the next logical step in our studies was to design the attachment position of a protein--for which X-ray structure is known--to the surface, and investigate its activity when immobilized at a planar surface.

Many attempts to construct protein-based biosensors have relied on natural proteins, since they have evolved to perform specific tasks in biological systems, which are beyond the capabilities of conventional chemical reactions [12]. However, for a system to become the basis of a successful technology it is important to characterize the properties of immobilized proteins on various surfaces and find the best combination of protein, surface linkers, and surface functionalities (surface energy), that produce optimal function and long-term stability.

Some progress has already been reported in this field. Pavlickova and coworkers developed a chip, which consists of a streptavidin sensor assembled on a gold surface, using nanoscale biotinilated SAM architectures [13]. Hu and coworkers nanografted *de novo *engineered S-824-C protein on gold [14], taking advantage of a technique invented by Liu [15]. Hoff and coworkers developed a nanoimprint-lithography (NIL) technique for producing high-contrast, high-resolution protein patterns [1].

A critical question, still unanswered, is how immobilization of a protein on a surface with particular chemical functionalities affects the protein structure and activity. In other words, can SAM chemistry mimic the natural environment of the protein? More open questions concern the conformational changes resulting from protein-surface interactions (post immobilization), and how they affect the activity of the immobilized protein. Answering these questions requires a study in which mixed SAMs are used to provide systematic changes in surface functionalities [16]. This is beyond the scope of this report. The key question we wish to answer here is whether designing the attachment site in the protein where surface attachment takes place will result in high protein activity. We selected the spontaneous formation of disulfide (S-S) bonds with the surface as immobilization reaction, and the protein we selected as the first model system is *Adenylate kina*se. The protein was attached to the surface by a SAM of hexane-1,6-dithiol.

**Figure 1:**
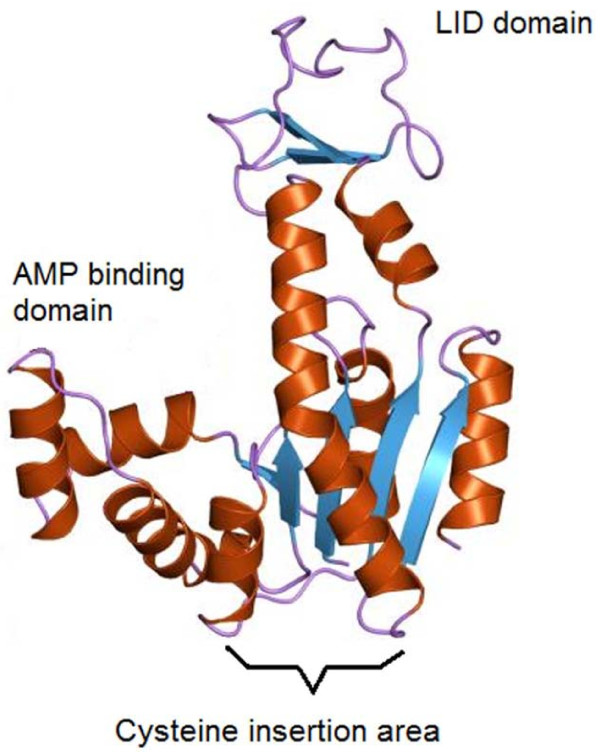
**The structure of Adenylate kinase with a general backbone view, showing the LID domain, the AMP-binding domain and the area of a mutation: substitution of the original residue at the 75th position by Cys**.

*Adenylate kinase *(PDB codename - 4ake) is a protein of 23.5 kDa, for which x-ray structure has been determined [17]. It is an essential phospho-transferase enzyme, which is responsible for recycling AMP in energetically-active cells [17]. The molecule, which consists of a single polypeptide chain, is folded into three domains: CORE, LID and AMP-binding (Figure 1), and catalyzes the transfer of a phosphate group from Mg-ATP to AMP. Although highly specific for AMP and dAMP, *Adenylate kinase *also showed detectible activity when ATP or dATP was replaced by a variety of ribonucleoside triphosphates [18]. It is a perfect model for studying the activity of a surface-immobilized protein, since its conformational properties are important, as large structural changes occur upon enzyme-substrate complex formation [18,19]. Thermodynamic properties, conformational changes [20] and possibly the forces that hold the protein in its active operating state, all can be studied systematically and in great detail. *Adenylate kinase*-based platforms can serve as fast and reliable systems for the detection of bacterial infections and the presence of dead cells.

**Figure 2:**
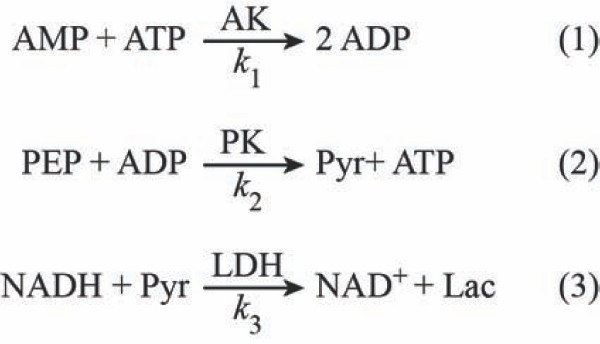
**A cascade reaction for calculating kinetics of AK, based on obtaining NAD^+ ^spectrum**. Originally proposed by Valero and coworkers. PEP-phospho-enol-pyruvate, PK-pyruvate-kinase, Pyr-Pyruvate, LDH-lactate dehydrogenase.

ATP is commonly thrown into extra-cellular space by bacteria [21,22] and NAD^+ ^is a common by-product of dead cells. With immobilized *Adenylate kinase *and a cascade of reactions, as shown by Valero and coworkers [23] (Figure 2), it is possible to detect reliably and quickly even the slightest amounts of by-products of ATP-based reactions and to calculate ATP levels from the optical absorbance of NAD^+^. The kinetic properties of *Adenylate kinase *are regarded as rapid and these, as well as the thermodynamic and biochemical properties have previously been closely studied and reported [20,23-25].

Thus, all that is needed is to compare the level of ATP or NAD^+ ^obtained to the physiologically normal value. If the NAD^+ ^level is higher than normal, it is also possible to remove one of the components (NADH or ATP) at a time and, by comparing the observed NAD^+ ^levels, determine whether the source was a potential bacterial infection or dead cells [18,21,22]. We believe that, because of the high activity of *Adenylate kinase*, the proposed concept can result in a biosensor that gives a result on the bacterial-infection/dead-cells test much faster than methods currently used in clinics. These methods are based on growing cultures for several hours and measuring the amount, if any, of CO_2 _emitted. Such tests take time - from two to 24 hours - whereas there are some flesh-eating bacteria that kill a healthy human in a day and a half.

Hexane-1,6-dithiol forms a SAM on gold surfaces with SH groups exposed at the SAM-air interface, since its short length makes surface attachment of both SH groups to form a loop less probable. This allows a protein containing a cysteine residue to be attached to the surface by a disulfide (S-S) bond, which is formed spontaneously, without any catalyst. The cysteine can be introduced to, or removed from the protein by standard molecular-biology techniques.

Native *Adenylate kinase *has only one natural cysteine, at residue 77 [20,24,25]. Additional cysteine moieties have been engineered into the molecule in order to insert fluorescent probes for determining intramolecular distances between fluorescent donors and acceptors in various stages of refolding with the use of FRET [20]. In such cases, the native cysteine was replaced by serine in order to avoid possible interactions with the newly-substituted cysteine. Such mutations did not alter the thermodynamic or activity properties [25]. Our experiments revealed that the native protein is not active when adsorbed either on bare gold or on the SAM, suggesting that the protein's operative domains are not available in the immobilized state, and further emphasizing the importance of controlling the protein attachment to the surface.

X-ray measurements [17] show that the protein's 75 position is at the "bottom" of the chain's three-dimensional structure, leaving the operative domains exposed to the environment. Hence, if immobilized at this position, the protein should be highly active. This is analogous to the self-assembly of *α*-Cyclodextrin derivatives on gold, where host-guest pairs for the nonmethylated cyclodextrin showed 1-2 orders of magnitude higher binding constants on surfaces than in solution [26]. Therefore, cysteine was inserted at the 75 residue of *Adenylate kinase *and the cysteine at residue 77 was replaced by serine, leaving one SH group for binding [25]. This mutant was used by Haas and coworkers and was shown to have a high refolding constant [21,22]. We assumed that when these protein molecules are arranged on the surface, any substrate molecule approaching an active site should trigger a reaction, and the reactivity should not be reduced upon immobilization.

## Methods

### Materials

Gold 9.999 (Sigma), 1,6-hexanedithiol, TCEP-HCl (tris-2(CarboxyEthyl)-Phosphine HydroChloride), TRIS buffer, AK(*Adenylate kynase*), and NADH were from Aldrich.

### Protein acquiring and purification

The mutated *Adenylate kinase *protein (Ser in 77 position, and Cys in the 75 position) was acquired from an *E. coli*. and purified by a procedure, described elsewhere [21]. The residue substitution mutagenesis--a routine procedure in molecular biology--was carried out using a *Stratagene's quick change mutagenesis kit*. The methylated and hemimethylated DNA was digested with *Dpn*. The procedure was performed according to the manufacturer instructions, available on-line [27].

### Gold substrate preparation

Gold evaporated and annealed on glass microscopic slide 3 × 1 × 1 under air pressure of 4 × 10^-6 ^Torr, using a Key Vacuum evaporator. The gold thickness was ~ 2000 Å.

### 1,6-Hexanedithiol SAM preparation

3 μL 1,6-hexanedithiol (Sigma, Aldrich) and 18 mL Ethanol ABS, AR, Anhydrous were mixed to form a 1 mM solution. The solution was degassed (nitrogen) for 10 min and a gold substrate was immersed in the solution. The system was degassed with nitrogen again for 30 min, and the flask was sealed hermetically, and was left overnight. The slide was then washed with ethanol, dried under a steam of nitrogen, and cut to pieces of approximately 6 × 6 mm to insure they fit into a standard 1 mm optical cuvette.

### *Adenylate-kinase *immobilization

Pieces of SAM coated slides were inserted into Ependorf tubes containing 2 mL of TRIS 20 mM solution, pH = 7.2. Under nitrogen, a 3 μL of TCEP was added to reduce possible oxidized SH groups, then *Adenylate kinase*, dissolved in TRIS buffer, approximately 1 mg/ml, was added in great excess (2 μL of protein-in-buffer solution). The solution was degassed with nitrogen for 15 min, closed, and left on a shaker overnight at +4°C.

**XPS measurements **were carried out using 5600 multi-technique system (Physical Electronics, USA) with a monochromatic Al K*α *source (1486.6 eV). The spectra were obtained in a high resolution mode, at pass energy of 11.75 eV and with 0.05 eV/step intervals.

**QCM measurements **were performed with a FLUKE 164T Counter, at 1.3 GHz frequency, and a gold-covered crystal of 0.392 cm^2 ^area. The surface coverage by either SAM or protein molecules was estimated using the Sauerbrey equation , where Δ*m *is the mass change, *f*_o _is the resonance frequency of the quartz crystal, *A *is the piezoelectrically active area, *ρ_q _*is the density of the quartz (2.648 g cm^-3^), and *μ_q _*is the shear modulus (2.947 × 1011 dyne cm^-3^) for AT-cut quartz.

## Results and Discussion

The water contact angle at the SH surface, determined using 1-2 μL droplets of distilled water, was 65-70°, in agreement with literature data [28]. The activity of the surface SH groups was determined by exposing the SAM to DTNB (5,5'-dithiobis-(2-nitrobenzoic acid), Elman's reagent). This reaction is rapid and stoichiometric. The same procedure was used to determine the activity of the SH groups in the protein. In both cases the appearance of a yellow color indicated that the SH groups were active and capable of forming S-S bonds. It was found that as-prepared SAMs were quickly oxidized (probably by ambient oxygen) to form surface S-S bonds. This oxidation is reversible and the disulfides could be reduced back to surface SH groups with the use of TCEP.

**Figure 3:**
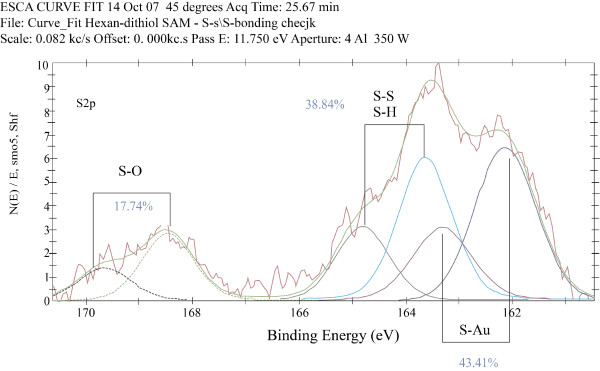
**The sulfur region in the XPS spectrum of hexane-1,6-dithiol SAM on gold**. Data were collected at 45°C, and acquisition time was 26 minutes.

Curve fitting in XPS measurements is a well-established and reliable procedure that uses a Gaussian-Lorentzian function, and provides an accuracy of two decimal digits [29]. The fitting was performed using 5600 Multi-technique system software (PHI, USA). The accuracy of fitting depends on the signal-to noise ratio for the measured curve, and in the present studies the S2p spectrum was rather noisy. Therefore the calculated areas are provided with accuracy up to an integer number: A_S-Au _= 43%, A_C-SH _= 39%, A_S-O _= 18%, (Figure 3). The S-Au bonds **(**A_S-Au _= 43%) are at Au/dithiol interface, and their S_2p _signal is measured after attenuation because of the SAM layer. On the other hand, the ω-SH group is at the SAM surface; hence, there is no attenuation of the S2p electrons.

To address the concentration of loops, the real quantity of the S-Au bonding should first be obtained, taking into consideration electron attenuation: L = exp(-d/λ) = 0.719, where L is the attenuation factor for the electrons coming through the monolayer, d is the layer thickness, assumed to be about 11 Ǻ (considering bond lengths and molecular tilt angle), and λ is the inelastic mean-free-path of electrons (33 Ǻ for this kind of a molecule) [30]. The real quantity of the S-Au bonding can be obtained by the equation A_(S-Au) real _= A_(S-Au) measured _: L = 43: 0.719 ≈ 60%. This 60% are composed of dithiol molecules, whose other end is SH (A_C-SH _= 39%) and bridged (B) thiol molecules (or "loops"), with both ends connected to Au. For convenience they will be marked as 2B, accounting for two molecular end-groups connected to Au. We did not use attenuation for the electrons coming from the loops. therefore if A_(S-Au) real _= S + 2B, then 60 = 39 + 2B, and B = 10.5 ≈ 11%. Thus, about 20% of adsorbed 1,6-hexanedithiol form loops.

X-ray photoelectron spectroscopy (XPS) showed clearly the two types of sulfur atom, one attached to the gold (thiolate) and the other (SH/S-S) at the ω-position for the hexane-1,6-dithiol SAMs (Figure 3) [31,32]. Notice that alkanethiolates in self-assembled monolayers on gold oxidize in air, in the dark, to form sulfinates and sulfonates and the thiolate gold interface (The S^- ^is more prone to oxidation than the SH), and gives rise to the S-O peak in the XPS spectra (~ 168.5 eV) [33-37].

**Figure 4:**
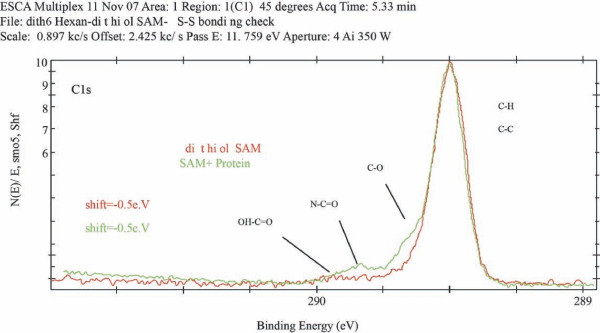
**Fitting of the XPS spectra of a carbon (1s) region for a sample containing a dithiol monolayer and a sample, containing immobilized protein**. Scale 0.897 kc/s. Offset 2.425 kc/s. Pass E 11.750 eV. Aperture 4 AI 350 W. Data were collected at 45°C, and acquisition time was 15 minutes

XPS spectra of the immobilized protein samples revealed significant increase in the carbon content, with the different protein carbons observed (Figure 4).

After the protein was chemically attached to the surface, XPS provided bonding percentage of S-Au and C-SH bonds, as A_(S-Au) measured _≈ 35%, and A_(C-S-protein) measured _≈ 28%, accordingly. If the attenuation in the dithiol layer for S2p electrons involved in S-Au bonding is taken into consideration, one obtains A_(S-Au) real _= A_(S-Au) measured _: L = 35: 0.719 ≈ 49%

There is also attenuation of electrons passing through the protein for both S-Au and C-SH electrons, and assumed to be the same for both of them, because of the large size of the protein, and hence is not considered here. The quantity of the forming loops is unchanged, 11%. The ratio of the A_(S-Au) real _to A_(C-SH) measured _for dithiol SAM and for dithiol SAM with protein (including bridged molecules) is 60:39 ≈ 1.54, and 49:28 ≈ 1.75 respectively. The ratio between the two numbers (1.54: 1.75 = 0.88) suggests that protein binding is accompanies by desorption of ~12% of 1,6-hexanedithiol molecules from the SAM in SAM. This loss might be understood if one assumes that the immobilized proteins are in a brush-like structure. Loading of more protein molecules at the surface results in less space available for each protein and thus the stretching of its chain to decrease intermolecular repulsion. such stretching can eventually result in breaking of the Au-S bond, which is relatively weak (~44 Kcal/mol) [38,39]. In fact, the same desorption phenomenon was observed for polystyrene brushes formed by surface-initiated living anionic polymerization of styrene using rigid lithiated biphenyl SAM surfaces as initiator. There, surface morphology studies by AFM showed holes in the brush and the desorbed chains accumulated on their edges [40].

**Figure 5:**
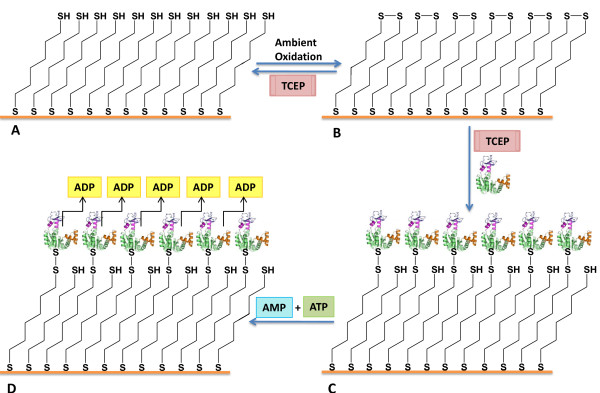
**The scheme of the overall AK-based platform preparation and test process**. (A) preparation of 1,6-hexane dithiol monolayer. (B) creation of multiple S-S bonds at the ω-position of the monolayer, as a result of oxidation. (C) addition of AK with low concentrations of TCEP for the prevention of aggregate formation. (D) testing the platform, according to the cascade of reactions, presented by Valero *et al.*

**Figure 6:**
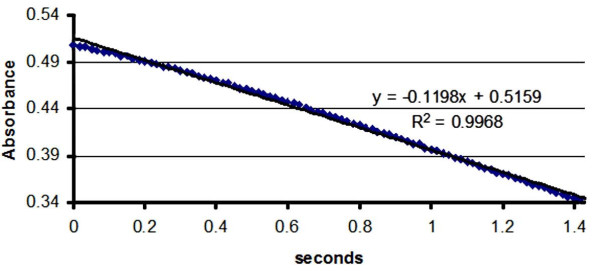
**Typical kinetics curve**.

Enzymatic activity tests for the immobilized *Adenylate kinase *were performed as described by Valero and coworkers [23]. Figure 5 shows a schematic diagram with a step-by-step graphical representation of the grafting of *Adenylate kinase *on the SAM surface and the study of its activity. Briefly, it consists of preparing the reaction medium from NADH, potassium acetate, MgCl_2_, AMP (previously treated with apyrase) [2], PEP(phospho-enol-pyruvate), pyruvate kinase, and L-lactate dehydrogenase imidazole/acetic acid buffer (pH 7.5). Immediately after the immobilized protein sample was added, the reaction was initiated by the addition of ATP and the kinetics was followed by measuring the disappearance of NADH at 37°C, following the 340 nm band in the absorption spectrum. This band results from a number of conjugated processes described in detail in the literature [23]. Figure 6 shows a typical kinetics curve.

The distribution of slopes of the linear portions of the kinetic curves of substrate disappearance, which provides information on *Adenylate-kinase *activity, ranged from -0.0732 to - 0.1198 (arbitrary units), with an average of -0.097. One of the samples was rechecked in a different solution, to ensure that the observed activity was from immobilized *Adenylate kinase *only and not from proteins that desorbed from the SAM surface. The fitted results of the samples in the first (-0.1198), and second (-0.1130) tests are well within experimental error. These numbers are averages, obtained from the kinetics slopes of at least five independent experiments. The enzymatic activity of 52 nanomoles of protein dissolved in TRIS buffer solution was measured, and the slope obtained was -0.1370. The method described by Valero [26] was used many times by the Haas group with excellent reproducibility. When experiments were carried out, in which one of the components was missing, no catalytic reaction was observed. This confirms that no loss of specificity results from immobilization on the surface.

The theoretical maximum number of protein molecules immobilized is about 2.3 × 10^-8 ^mole/sample if a hexagonal arrangement of molecules is assumed, and 1.62 × 10^-10 ^for cubic arrangement. It was suggested by Patolsky [41], Granot [42] and Willner [43], that globular proteins tend to pack in homogeneous cubic-like form. However, the actual form of packing is not critical, since the controlling factors are the number of immobilized molecules, and the significant free space around the immobilized protein molecules, as is discussed below.

To prove that the observed activity came exclusively from immobilized protein, and to obtain an experimental value for the amount of protein on the surface, a series of QCM experiments were performed.

The average frequency of the crystal that was used, at ambient temperatures, was 8.9869614 MHz, and the addition of hexane-1,6-dithiol to the crystal resulted in a measured frequency of 8.9868661 MHz. The change in frequency (*Δf*) was 95 Hz. Protein attachment resulted in an average frequency of 8.9864262 MHz. *Δf *was approximately 43.99 Hz. According to the Sauerbrey equation, at the abovementioned basic sensitivity of the crystal, a change in frequency of 1 Hz indicates the addition of a mass of 1 ng/cm^2^. Since the effective area of crystal is lower than 1 cm^2 ^(0.392 cm^2^), the addition of the protein mass could be represented as . If a crystal area of 1 cm^2 ^is assumed, and with a knowledge of the molecular weight of *Adenylate kinase *- 24000 g/mol - the calculated number of moles for the protein is  moles. Multiplying the calculated number of moles by Avogadro's number gives the number of molecules per cm^2^, which is approximately 2.82 × 10^12^. Since the effective area of the crystal is 0.392 cm^2^, the actual number of protein molecules on the crystal surface was ~ 1.1 × 10^12^. The total area of the protein molecules was estimated by multiplying previously estimated number of molecules by the reported cross-sectional area of a single protein molecule [19].

The area covered with protein molecules, estimated by dividing the substrate surface area by the reported cross-sectional area of the protein, was approximately 0.2 cm^2^, which is ≤60% of the crystal surface area and suggests a homogeneous protein monolayer [41-43].

**Figure 7:**
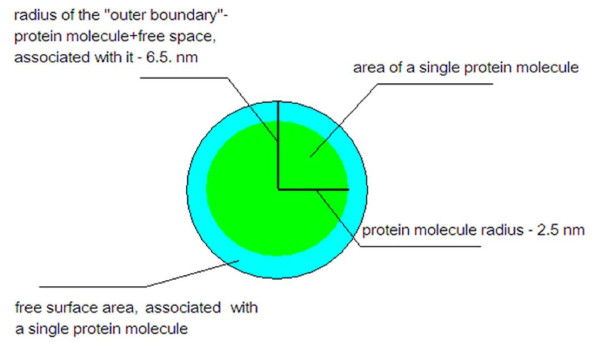
Schematic representation of basic model, used in calculation of free surface space, associated with a single protein molecule on surface

If the molecules are dispersed homogeneously over the surface and cover only maximum 60% of it, 40% of the surface is empty. Since this ratio is 40/60 = 0.67, for every nm^2 ^covered by protein molecules, there is 0.67 nm^2 ^of free surface. Since the protein is 5 nm in diameter [17], it occupies 19.63 nm^2 ^(Figure 7). Therefore, the free area associated with each protein molecule should be, on the average, 19.63 × 0.67 = 13.15 nm^2^. If one assumes that the protein occupies a circle of diameter 5 nm, concentric with a larger circle, whose area is that occupied by protein, combined with the area of free space around it -- 32.78 nm^2 ^-- then the diameter of the outer circle should be 6.5 nm. This means that the distance between the protein and the boundary of the larger circle is 1.5 nm and the *average *distance between two protein molecules is 3 nm.

This results in a significant free volume that should allow easy opening of the LID and hence efficient catalysis and high reactivity. In the case presented, the effective coverage is even less than 60%, which increases the average distance between protein molecules. The fact that only about 60% coverage is obtained, might suggest that in addition to repulsion forces between the surface-attached proteins, molecular motion could be an important factor in determining the coverage.

**Figure 8:**
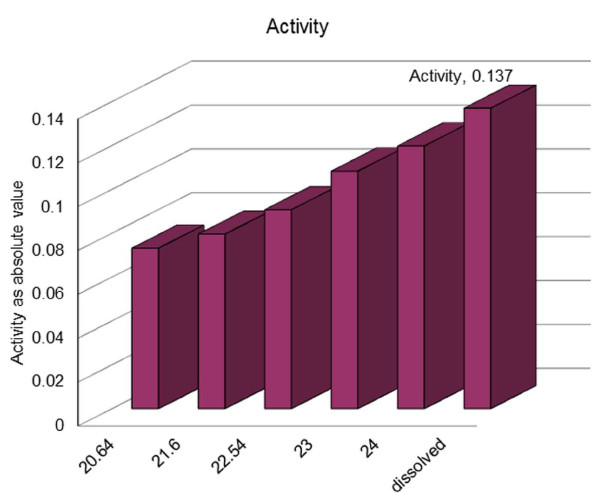
**Absolute values of enzyme activity (a.u.) for samples with different surface areas (mm^2^)**.

According to the results of QCM experiments, the amount of adsorbed surface protein, which produced activity equal to that of a nanomole of non-immobilized protein, was 10^-11 ^mole. This surprising result is encouraging, and supports our initial hypothesis that, when the system is properly designed, the activity of surface-adsorbed proteins can be higher than that in solution. Indeed, QCM measurements suggest that the activity of an *Adenylate kinase *protein attached through an S-S bond to a gold surface with the use of a hexane-1,6-dithiol SAM is *about 100 times higher *than that of the protein molecule in solution. We note that enzymatic activity depends on sample size (the amount of immobilized protein), as shown in Figure 8. Since it is difficult to achieve high precision with such small samples, one must consider up to 10% error in the sample dimension. This means that the error in the calculated amount of the immobilized protein could be up to 10%, and the average observed activity is *90 times or more higher *than that of the protein molecule in solution.

While immobilization could increase protein activity[44], the high activity observed in this case proves that the activity of the immobilized protein results from the molecular design of the protein. With the cysteine at the 75 residue forming the S-S bond with the SAM, the protein at the surface is positioned with its active site facing outwards, and hence fully available, in analogy to the Cyclodextrin-SAM system [26]. In addition, the free volume resulting from 60% coverage is advantageous, because it provides the space needed for active-site operation, *i.e*. for the LID and AMP-binding domain movements. This means that any substrate molecule passing close enough to the active site has a high probability of being converted to product.

Finally, and probably most interesting is that, while attempts to immobilize the mutated protein directly to the gold surface resulted in no activity, connecting it to a hexane-1,6-dithiol SAM, *via *an S-S linkage, was enough not only to maintain functionality, but also to exhibit very high reactivity. The lack of activity for the direct immobilization on gold was unexpected, since cysteine-containing engineered IgG-binding protein on a gold surface retained the same IgG-binding activity as the native protein [44]. However, in that case, the antigen-binding activity of immobilized antibody molecules on a gold surface was about 4.3 times higher than that of physically-adsorbed antibody molecules. Our conclusion is that if the formation of S-S bonds cannot take place, the mutated *Adenylate kinase *might be physically adsorbed on the bare gold surface with its active site hindered from contact with the solution.

It is also possible that the immobilized *Adenylate kinase *forms H-bonds with remaining surface SH groups, which, weak as they may be, affect protein conformation. For example In crystalline 2-mercaptobenzoic acid, the S-H groups were found to form an infinite S-H ⋯ S-H ⋯ S-H hydrogen-bond chain [45].

Finally, it is possible that interactions between the adsorbed protein and the underlying thiolate/Au system affect its structure and reactivity. To understand the mechanism that might be behind such interaction we first point to a study by Miller and Abbot which showed that the hexadecane contact angles on alkanethiolate SAMs on gold, taken in air, are measurably influenced by van der Waals forces that act between the liquid and the metallic substrate (through the SAMs) [46]. This effect decrease with increasing thiolate-alkyl chain length. We used surface-potential measurements to study thiolate SAMs on gold and showed that image charges are formed in the gold because of thiolate adsorption [47]. Thus, it is possible that the direction is space of bond dipoles in the protein is affected by interactions with the underlying image dipole structure.

We recognize that these final arguments require systematic studies, part of which are being conducted right now, but we have decided to discuss those issues with the hope that they will incite more studies, especially because of the importance of immobilizing proteins on magnetic and non-magnetic nanoparticles [7].

## Conclusions

To the best of our knowledge, immobilization of a protein by the method presented here, with the resulting high enzymatic activity, has never been reported. Clearly, more work needs to be done, including immobilization of other enzymes with the use of the unique route we have developed. Once established as a general method, it could represent an important step in the immobilization of proteins for a wide range of applications, especially after systematic studies of enzyme activity as a function of the distance from the thiolate/gold interface have been carried out.

The developed platform could be used in further R&D to develop a rapid and precise medical sensor for bacterial infections or dead cells presence. Systems based on proteins-on-gold technology are ecologically clean, and gold is an FDA-approved material. The prospects of having immobilized proteins with activities as high as in solution - and even higher - are very wide, and Jiang and coworkers suggest that palladium could replace gold as a platform for biotechnological applications [48]. There are a number of benefits for such a technology, among them high resolution of operation, generality, speed of operation, small surface area required, and ease of disposal. These attributes counterbalance cost, when one considers the many platforms on which protein technology could be applied.

The primary challenge in developing such a technology is that every protein used in an immobilized-protein-based application must be tested for activity while immobilized. Proteins can be acquired and purified in relatively large quantities, just as it has been done with *Adenylate kinase*. There are many advantages of protein-based technology over traditional ones, but the greatest is that with protein-based devices the action is much more precise, and the yield in multi-step processes could be up to 50% higher, with almost no side products.

Due to the success of the method, presented here, it was decided to continue a research project of a biosensor by transferring it to a high aspect ratio platform - nanotubes.

## List of abbreviations

SAM: self assembled monolayer; MEMS: Micro-Electro-Mechanical Systems; FRET: Fluorescence resonance energy transfer; NIL: nanoimprint-lithography; AK: *Adenylate kinase*; PDB: Protein data bank; PK: pyruvate kinase; PEP: phospho-enol-pyruvate; AMP: adenozin monophosphate; ADP: Adenosin diphosphate; ATP: Adenosine triphosphate; NAD: Nicotinamide adenine dinucleotide, XPS: X-ray photoelectron spectroscopy; QCM: Quartz crystal microbalance; DNA: Deoxyribonucleic acid; R&D-research and development.

## Competing interests

The authors declare that they have no competing interests.

## Authors' contributions

MI carried out molecular biology and biochemical studies, designed and developed the AK-based platform, performed SAM covered substrates preparation and characterization and QCM data acquisition and analysis, participated in the design of study and helped to draft the manuscript. AU supervised the study and participated in its design and coordination. Oversaw the drafting of the manuscript. DR performed SAM covered substrates preparation. FP have been involved in revising the manuscript critically for important intellectual content. EH have been involved in the design and coordination of the study as well as in drafting the manuscript and revising it critically for important intellectual content. All authors read and approved the final manuscript.
